# Ecto-nucleotidases Activities in the Contents of Ovarian Endometriomas: Potential Biomarkers of Endometriosis

**DOI:** 10.1155/2014/120673

**Published:** 2014-09-03

**Authors:** Laura Texidó, Claudia Romero, August Vidal, José García-Valero, M. Eulalia Fernández Montoli, Núria Baixeras, Enric Condom, Jordi Ponce, Amparo García-Tejedor, Mireia Martín-Satué

**Affiliations:** ^1^Departament de Patologia i Terapèutica Experimental, Facultat de Medicina, Universitat de Barcelona, Campus de Bellvitge, L'Hospitalet de Llobregat, 08907 Barcelona, Spain; ^2^Institut d'Investigació Biomèdica de Bellvitge (IDIBELL), Barcelona, Spain; ^3^Servei d'Anatomia Patològica, Hospital de Bellvitge, Barcelona, Spain; ^4^Departament de Biologia Cel*·*lular, Facultat de Biologia, Universitat de Barcelona, Barcelona, Spain; ^5^Servei de Ginecologia, Hospital de Bellvitge, L'Hospitalet de Llobregat, 08907 Barcelona, Spain

## Abstract

Endometriosis, defined as the growth of endometrial tissue outside the uterus, is a common gynecologic condition affecting millions of women worldwide. It is an inflammatory, estrogen-dependent complex disorder, with broad symptomatic variability, pelvic pain, and infertility being the main characteristics. Ovarian endometriomas are frequently developed in women with endometriosis. Late diagnosis is one of the main problems of endometriosis; thus, it is important to identify biomarkers for early diagnosis. The aim of the present work is to evaluate the ecto-nucleotidases activities in the contents of endometriomas. These enzymes, through the regulation of extracellular ATP and adenosine levels, are key enzymes in inflammatory processes, and their expression has been previously characterized in human endometrium. To achieve our objective, the echo-guided aspirated fluids of endometriomas were analyzed by evaluating the ecto-nucleotidases activities and compared with simple cysts. Our results show that enzyme activities are quantifiable in the ovarian cysts aspirates and that endometriomas show significantly higher ecto-nucleotidases activities than simple cysts (5.5-fold increase for ATPase and 20-fold for ADPase), thus being possible candidates for new endometriosis biomarkers. Moreover, we demonstrate the presence of ecto-nucleotidases bearing exosomes in these fluids. These results add up to the knowledge of the physiopathologic mechanisms underlying endometriosis and, open up a promising new field of study.

## 1. Introduction

Endometriosis is a chronic condition characterized by the presence of endometrial tissue (stroma and glands) outside the uterine cavity, mostly on pelvic peritoneum and ovaries. It affects up to 15% of women of reproductive age and its prevalence is rising. It is an inflammatory, estrogen-dependent complex disorder, with broad symptomatic variability, pelvic pain, and infertility being the main characteristics. Both symptoms are thought to be the result of an excessive inflammatory environment not only within the pelvis but also in the eutopic endometrium, affecting implantation [1, 2].

It is one of the principal causes of infertility in women. Late diagnosis is one of the main problems of this pathology, taking between five and ten years from detection of the first symptoms. Therefore, there is a need to identify biomarkers for early diagnosis of endometriosis [3].

Ovarian endometriomas occur in 17–44% of patients affected by endometriosis [4] and account for 35% of benign ovarian cysts. Endometriomas are also called endometriotic or “chocolate cysts” because of the internal fluid blood derived dark color [5]. Although the risk of malignant transformation is low, 0.6–0.8% [6], surgery has become the gold standard therapy. Nowadays, laparoscopic cystectomy is the most extended treatment for endometriomas, although fertility could be afterwards compromised. Some studies have reported a lower ovarian reserve after excision of ovarian endometriomas due to incidental excision of normal ovarian tissue together with the endometrioma wall [7, 8]. There is also evidence of a significant postoperative decrease in circulating Anti-Müllerian hormone, a commonly used biomarker for ovarian reserve quantification, suggesting a negative impact on ovarian reserve after the excision of endometriomas [8]. Therefore, a new minimally invasive interventional technique was implemented to treat low risk malignancy ovarian cysts, including endometriomas, the ultrasound-guided aspiration followed by sclerosis, which seems to have even a low recurrence risk (0–15%) [9–13]. The sclerosis does not seem to have consequences on the number of pregnancies, term pregnancies, abortions, extracted oocytes, embryo quality, and hormonal levels if compared with infertile women without ovarian cysts [11, 14].

The analysis of the contents of endometriomas can help identify new biomarkers for this pathology. For example, endometriotic cysts contain significantly more iron than other ovarian cysts; this has been suggested as a possible cause of carcinogenesis in endometriomas through iron-induced persistent oxidative stress [15]. Proteomic analysis of follicular fluid of women with endometriosis has also pointed to an altered immunologic function possibly related to infertility [16].

Purinergic signalling, the group of biologic effects exerted by extracellular nucleotides such as ATP and nucleosides such as adenosine through specific receptors, is an important regulatory mechanism in a wide range of inflammatory conditions [17, 18]. ATP is a “danger signal” released under tissue stress conditions, such as necrosis, apoptosis, hypoxia, and inflammation and is rapidly converted into adenosine, which protects cells and tissues from excessive inflammation and immune-mediated damage. The effects transduced by both molecules are thus often opposite, and the resulting cellular responses are the consequence of the ratio of ATP (and ADP) to adenosine concentrations, which is mainly controlled by ecto-nucleotidases, key enzymes in the purinergic signalling [19]. The most well-characterized ectonucleotidase pathway involves the sequential action of CD39 (ectonucleoside triphosphate diphosphohydrolase-1, NTPDase1), which converts extracellular ATP (or ADP) to AMP, and CD73 (ecto-5′-nucleotidase), which generates adenosine from AMP [20–23]. We have previously characterized the expression of endometrial ecto-nucleotidases, demonstrating changes along the cycle and in menopause [24, 25]. We have also demonstrated that CD39 and CD73 are highly expressed in endometrial cancer [26], contributing to the increased immunosuppressive levels of extracellular adenosine found in tumor microenvironment.

Exosomes are small, secreted membrane vesicles (30–100 nm in diameter) of endocytic origin, produced by many different cell types that are formed by the fusion of multivesicular endosomes with the plasma membrane. They are thought to play an important role in intercellular communication, for example, during immune cell-cell interaction, and have been identified in biological fluids, such as plasma and urine, as well as in cell culture supernatants [27, 28]. The presence of the ecto-nucleotidases CD39 and CD73 has been previously demonstrated in cancer exosomes [29–31], contributing to the adenosine production that suppresses T-cell function.

In the present work, we aimed to determine whether ecto-nucleotidases activities (ATPase, ADPase, and AMPase) are present and measurable in the ultrasound-guided aspirated contents of ovarian cysts and whether these activities are altered in endometriotic cysts compared with simple cysts, affecting the inflammatory condition of endometriosis. Our goal is to evaluate their potential as biomarkers for endometriosis and to contribute to the knowledge of the physiopathologic mechanisms underlying endometriosis.

## 2. Materials and Methods

### 2.1. Samples

The ethical principles of this study adhere to the Declaration of Helsinki, and all the procedures were approved by the ethics committee for clinical investigation of Bellvitge Hospital. A prospective cohort of 27 adnexal cysts with a low risk of malignancy, including 14 endometriomas and 13 ovarian simple cysts, was studied at the Gynecology Service of Bellvitge Hospital (Barcelona, Spain) between March 2013 and April 2014.

Inclusion criteria in the study for patients were (1) aged >18 years old; (2) ultrasound features predictive of a low risk of cyst malignancy: smooth walls, no solid component, maximum diameter between 40 and 100 mm, no more than one thin septation (<2-3 mm), and no internal flow on color Doppler imaging in the case of bilocular cysts, according to IOTA criteria [32]; (3) cyst persistence from diagnosis ≥6 months; (4) tumor marker Ca 125 < 200 IU/mL and Ca 19.9 < 35 IU/mL; and (5) written informed consent from the patients.

For ultrasound-guided aspiration, following the abdomen, or the vagina, disinfection, a sterilized 17 G spinal needle (BD Medical Franklin Lakes, NJ Becton, Dickinson and Company) was aimed at the center of the cyst under direct ultrasound guidance and the contents was aspirated. When thick highly dense intracystic fluid was present, in order to facilitate the aspiration, saline dilutions were performed. Dilutions made were then considered in the final calculations. Then, several intracyst saline washes were performed until clearance, followed by the instillation of ethanol as sclerosing agent. The volume of instilled ethanol was 2/3 of the aspirated volume of the endometrioma and always less than 100 mL. Ethanol was left inside the cyst for 15 min and then removed and washed out again with a saline solution. The intracystic fluids were analyzed cytologically at the Pathology Service of Bellvitge Hospital to confirm the absence of atypical cells.

Patients were divided into two groups following the ultrasound features and internal color fluid: (a)* endometriomas group* that had homogeneous appearance involving diffuse internal echoes on a hypoechoic background and contained blood color fluid and (b)* simple cysts group* that had an anechoic structure and contained clear serous fluid.  Table 1 includes the descriptive statistics of the two groups of patients, including the age and the size and the volume of the cysts.

An aliquot of each sample was maintained on ice until usage for exosome enrichment. The rest was aliquoted and stored at −80°C.

### 2.2. Exosome Enrichment

Endometriomas and simple cysts fluid samples were centrifuged at 3000 ×g for 15 min to remove cells and cell debris, and supernatants were transferred to sterile tubes. 250 *μ*L of each supernatant was mixed with 63 *μ*L of ExoQuick Exosome Precipitation Solution (System Biosciences, Mountain View, CA, USA) by inversion, and the mixture was refrigerated overnight at 4°C, following the supplier's instructions. Samples were then centrifuged at 1500 ×g for 30 min. After centrifugation, the exosomes appeared as a beige or white pellet at the bottom of the tube. Supernatant was aspirated and the residual ExoQuick solution was spun down by centrifugation at 1500 ×g for 5 min. All traces of fluid were removed by aspiration and exosome pellets were resuspended in two different buffers. For Western blot analysis, exosome pellets were resuspended in 75 *μ*L of a buffer containing 10 mM Tris-HCl pH 7.4, 150 mM NaCl, and 1 : 100 proteases inhibitor cocktail (Sigma-Aldrich, Saint Louis, MO, USA). Alternatively, for ecto-nucleotidases activity assays, exosome pellets were resuspended in 75 *μ*L of enzyme assay buffer (160 mM Tris-HCl pH 7.4, 10 mM CaCl_2_). Protein concentration in exosome samples was determined by the method of Bradford [33]. Samples were stored at −20°C until usage.

### 2.3. Western Blotting Analysis

15 *μ*g of protein was heated at 95°C for 3 min, loaded onto any kD Mini-PROTEAN TGX Precast Gel (Bio-Rad, Hercules, CA, USA), and transferred to polyvinylidene fluoride (PVDF) membranes using the Trans-Blot Turbo Transfer System (Bio-Rad). Blots were blocked with Tris-buffered saline with 0.1% Tween-20 (TBS-T) containing 5% nonfat dry milk for 1 hour at room temperature (RT). Membranes were then incubated overnight at 4°C with the following antibodies: exosomal marker antibodies CD9, CD63, CD81, HSP70 (diluted 1 : 1000) from the ExoAB-KIT-1 Western blot antibody detection kit (System Biosciences), anti-CD39 (clone BU61, diluted 1 : 500), and anti-CD73 (clone 4G4 from Abcam, diluted 1 : 50). Blots were then washed with TBS-T and incubated with the appropriate HRP-conjugated secondary antibody (System Biosciences, dilution 1 : 20,000) for 1 hour at RT. Membranes were then incubated with Amersham ECL Western Blotting Detection Reagents (GE Healthcare, Pittsburgh, PA, USA) for 1 min and exposed on X-ray films.

### 2.4. Nucleotidase Activity Assays

Nucleotidase activities (ATPase, ADPase, and AMPase) were determined in flat-bottom 96-well plates by measuring the amount of liberated inorganic phosphate (Pi) using the malachite green colorimetric assay [34]. All the samples were used for these assays. The incubation mixture contained 160 mM Tris–HCl pH 7.4, 10 mM CaCl_2_, 5 mM levamisole, as alkaline phosphatases inhibitor, and 1 mM ATP, ADP, or AMP as substrates in a final volume of 150 *μ*L. The assay was initiated by adding the sample, either the fluid directly (5 *μ*L for ATPase and ADPase assays and 10 *μ*L for AMPase assay) or 50 *μ*g of the isolated exosome fraction. Alternatively, increasing amounts of exosomes were added to the well to assess if ectonucleotidase activity depended on the exosome dose (5, 10, 15, 20, 30, and 50 *μ*g). After 20 minutes of incubation at 37°C, 50 *μ*L of malachite green solution was added to each well and 10 and 20 minutes later, the OD_620_ was measured and recorded. Incubation times and protein concentrations were chosen to ensure the linearity of the enzymatic reaction. KH_2_PO_4_ was used as a Pi standard. Controls to determine nonenzymatic Pi accumulation were performed by incubating either the samples in the absence of the substrate or the substrate alone. Each experiment was performed three times and in each experiment samples were analyzed in triplicate. Enzyme activity was expressed as pmols Pi/min/*μ*L in the case of the whole fluids and as pmol Pi/min/*μ*g for exosomes.

### 2.5. Electron Microscopy Studies

For morphological analysis, whole-mount electron microscopy preparations of exosomes were obtained as previously described by Théry et al. [35]. In brief, exosome-containing pellets were resuspended in 2% paraformaldehyde in 100 mM phosphate-buffered saline (PBS). 5 *μ*L of resuspended pellets was deposited on formvar-coated grids and incubated at RT for 20 min. After thorough rinses, the samples were postfixed in 1% glutaraldehyde for 5 min and then contrasted in a solution of 2% uranyl acetate, 150 mM oxalic acid pH 7. Then the grids were transferred to a drop of 1.8% methyl-cellulose, 0.4% uranyl acetate for 10 min on ice. Finally, the grids were removed with stainless steel loops and air-dried for additional 10 min.

Alternatively, and in order to obtain thin sections, the paraformaldehyde-fixed exosome-containing pellets were postfixed in 2.5% glutaraldehyde and 2% paraformaldehyde in 100 mM PBS, dehydrated in a graded ethanol series, and embedded in the Spurr low viscosity resin. Ultrathin sections (50–60 nm) from exosome-enriched pellets were cut with a diamond knife in an ultramicrotome UltracutE (Leica Microsystems). After being picked up on copper grids, the ultrathin sections were stained with uranyl acetate and lead citrate. Finally, exosomes were observed with a JEM-1010 transmission electron microscope (JEOL, Japan), and the images were recorded with a Bio Scan 792 camera (GATAN, Germany).

### 2.6. Statistical Analysis

Statistical analysis was performed using SigmaStat 3.2 software (SPSS Inc., Chicago, IL, USA). Values are reported as the mean ± S.E.M. Student's *t*-test was used to compare the means of two independent groups of normally distributed data.

## 3. Results and Discussion

Most of the attempts to identify peripheral biomarkers of endometriosis have been focused on their presence in plasma, serum, or urine [3]. However, the study of endometriomas contents has been proven to be very informative [15, 16]. In the present work, we have evaluated the ectonucleotidase enzyme activities present in endometriomas in comparison with ovarian simple cysts.

**Figure 1:**
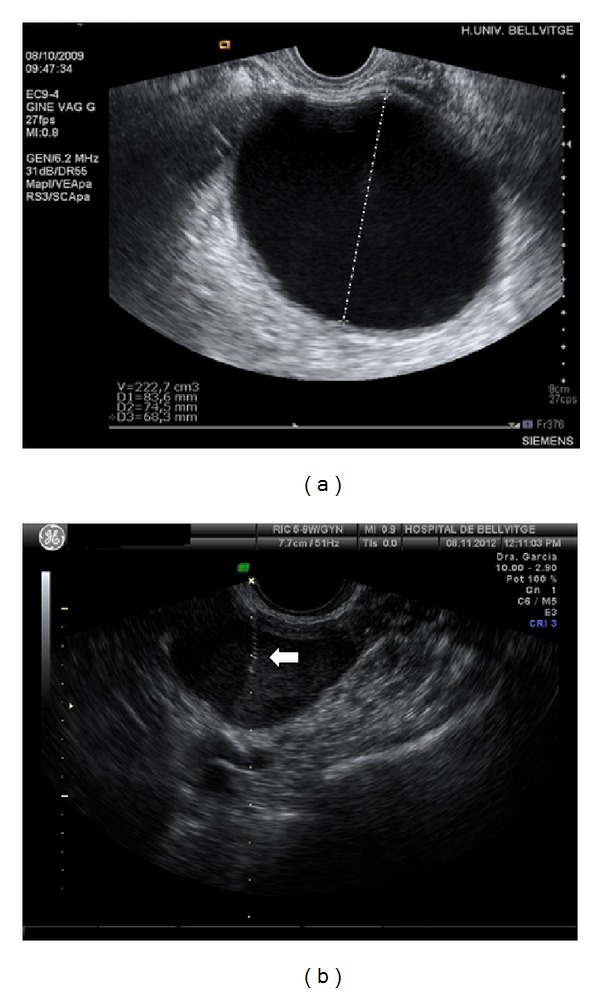
Transvaginal ultrasound-guided aspiration of an endometrioma. (a) Endometrioma measured before aspiration. Dotted line indicates one of the diameter measurements. (b) Endometrioma being aspirated. The arrow indicates the end of the needle inside the cyst. Note the reduction in the volume after aspiration.

Echo-guided aspiration was the technique used to remove the fluid contained in endometriomas and simple cysts. Immediate ethanol sclerosis was performed to chemically destroy the cyst lining and to prevent its regrowth.  Figure 1 shows the ultrasound picture of an endometrioma before and during the aspiration of its contents.

**Figure 2:**
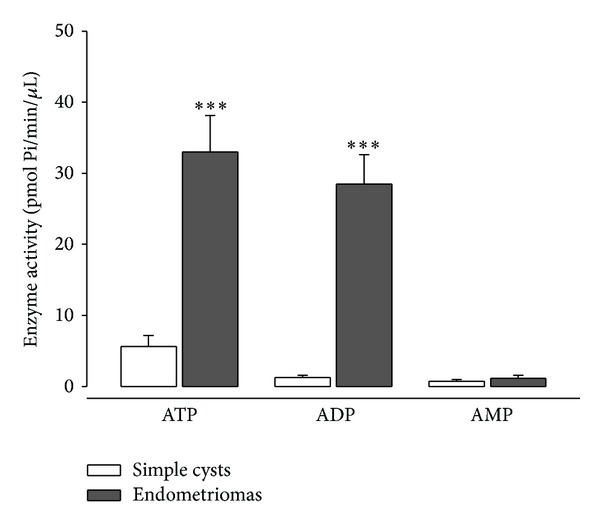
Enzyme activity in fluids from simple cysts and from endometriomas. Ectonucleotidase activity, represented as pmoles of generated Pi, in fluids from simple cysts (white columns) and from endometriomas (gray columns) in the presence of ATP, ADP, or AMP as substrate. ATPase and ADPase activities are significantly higher in endometriomas than in simple cysts (***P < 0,001), while AMPase activity was barely detectable and differences could not be determined.

Aspirated fluids were then used for enzyme activity studies. Ecto-nucleotidases activities (ATPase, ADPase, and, to a lesser extent, AMPase) were present and quantifiable in the aspirated fluid of both endometriomas and ovarian simple cysts (Figure 2). Endometriomas aspirates showed significantly higher (*P* < 0,001) ATPase and ADPase activities (mean ± SEM, 38,96 ± 5,11 and 33,68 ± 3,95 pmol/min/*μ*L, resp.) than simple cysts (7,19 ± 1,88 and 1,55 ± 0,4 pmol/min/*μ*L, resp.). Low activity rates were detected with AMP as substrate in the aspirated fluid and although endometriomas displayed higher AMPase activity (1,28 ± 0,46 pmol/min/*μ*L) than simple cysts (0,87 ± 0,25 pmol/min/*μ*L), differences were not significant. The same results were also obtained when the activities were calculated per *μ*g of protein in the fluid, with even higher activities in endometriomas (data not shown); however, since it is known that endometriotic cysts differ from simple cysts in protein contents [16], results are shown here as Pi generation per unit of volume. This is the first time that ecto-nucleotidases have been studied in ovarian cysts, although these enzyme activities have been previously characterized in other fluids such as pancreatic juice [36] and plasma [37], where there is a correlation between their activity and different inflammatory pathologies [38].

**Figure 3:**
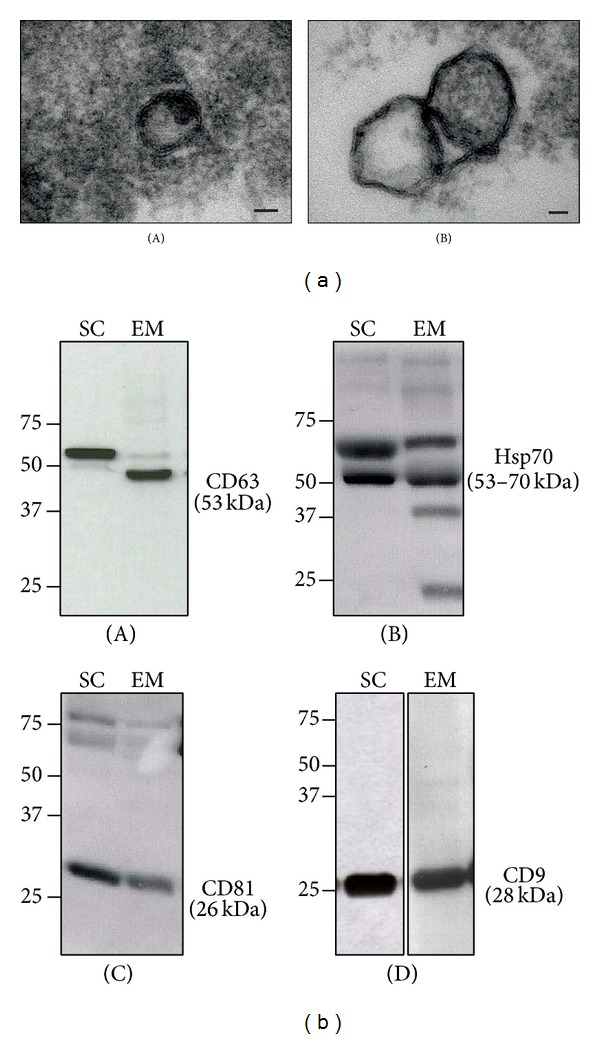
Exosome characterization. (a) Representative TEM images of exosomes isolated from endometriomas (bar = 25 nm). (b) Western blot with four different exosome markers: CD63 (A), Hsp70 (B), CD81 (C), and CD9 (D) of exosome-enriched fractions from simple cysts (SC) and from endometriomas (EM). The figure shows one representative experiment of each type of the sample. Molecular weight markers in kDa are indicated at the left of each image.

Since to perform the enzyme activity assays, aspirated fluids were previously centrifuged to remove cells and cell debris, the resulting activities could be attributed either to soluble enzymes or to membrane bound ecto-nucleotidases present in extracellular membrane vesicles such as microvesicles and exosomes that were not removed by the initial centrifugation. Therefore, we decided to perform exosome enrichment to assess their presence and to determine if they had ectonucleotidase activities. To our knowledge, this is the first study demonstrating the presence of exosomes in the fluids contained in ovarian cysts. Electron microscopy analysis confirmed the size and morphology of exosomes (Figure 3(a)). Western blots using antibodies against four exosome markers (CD9, CD63, CD81, and Hsp70) demonstrated the presence of these structures in 100% of endometriomas and in 46% of simple cysts aspirates (Figure 3(b)). From these experiments, we conclude that, among the markers used, CD9 is the most suitable for exosome demonstration in the aspirated fluids of ovarian cysts, since it was present in all exosome samples analyzed. CD9 has been already demonstrated to be a good exosome marker for epithelial endometrial cells [39, 40]. All the samples expressed, together with CD9, at least one more of the other exosome markers assayed. Interestingly, using the anti-CD63 antibody, the predominant band in endometriomas was of a lower molecular weight than in simple cysts. A possible explanation is the alternative splicing already described for the gene encoding CD63 that results in multiple transcript variants encoding different proteins. Since this result was consistent in all the samples studied, we propose to further study its presence in exosomes from other origins in endometriosis patients, to establish if it could be used as biomarker for the pathology.

**Figure 4:**
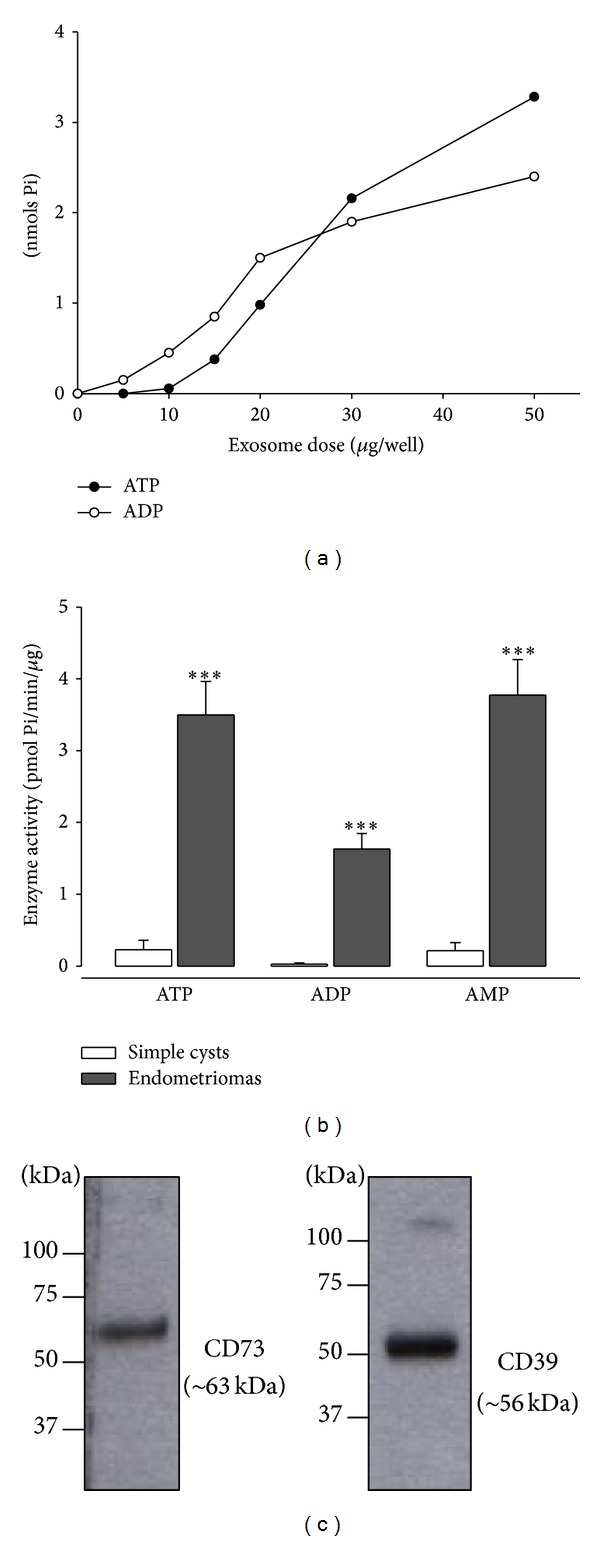
Enzyme activity in exosome-enriched fractions from simple cysts and from endometriomas. (a) ATPase (black circles) and ADPase (white circles) activities of increasing amounts of exosomes from endometriomas (0, 5, 10, 20, 30, and 50 *μ*g). (b) Ectonucleotidase activity, represented as pmoles of generated Pi, in exosome-enriched fractions from simple cysts (white columns) and from endometriomas (gray columns) in the presence of ATP, ADP, or AMP as substrate. All the activities are significantly higher in endometriomas than in simple cysts (***P < 0,001). (c) Western blot with anti-CD39 and anti-CD73 antibodies of exosome-enriched fractions from an endometrioma. Molecular weight markers in kDa are indicated at the left of each image.

Enzyme experiments performed with exosome-enriched fractions demonstrated that these samples retain the ectonucleotidase activity (ATPase, ADPase, and AMPase) displayed by the whole fluid. Moreover, these activities are dependent on the sample dose, as shown in Figure 4(a), in line with previous results obtained with exosomes from other origins that express active CD39 and CD73 [29]. For ATPase and ADPase, maximal activity was found with 50 *μ*g of exosomes. AMPase activity, which was barely detectable in the whole fluid, was present in purified exosomes, though only at high concentrations (50 *μ*g), indicating that this activity in the whole fluid is mainly due to the enzymes bearing exosomes. On the contrary, ATPase and ADPase activities would be related to both exosome membrane bound and soluble enzymes.  Figure 4(b) shows the enzyme activity using ATP, ADP, and AMP as substrates of exosomes from simple cysts and from endometriomas. These activities were significantly higher in endometriomas than in simple cysts. Western blot analysis demonstrated the presence of CD39 and CD73 in these samples (Figure 4(c)).

Our results reinforce the emerging evidence linking inflammation to endometriosis pathophysiology, where evasion of the immune surveillance seems to be crucial in the initial establishment and subsequent development of displaced endometrial tissues [41]. In this sense, increased sequentially acting ectonucleotidase activities would contribute to the maintenance of the suppression of local immune responses necessary for the disease progression, by regulating extracellular ATP and rising extracellular adenosine levels. We propose to detect their presence in other more accessible biological fluids, a fact that, together with the simplicity of their detection, would make them promising endometriosis biomarkers.

## 4. Conclusions

Our work contributes to the knowledge of endometriosis by providing new data on the fluid composition of endometriomas. Ecto-nucleotidases activities are quantifiable in the ovarian cysts aspirates, and they are significantly increased in the contents of endometriomas compared with simple cysts. Moreover, the identification of ecto-nucleotidases bearing exosomes in ovarian cyst contents opens the field for a broad range of proteomics and genomics studies.

## Figures and Tables

**Table 1:** Descriptive statistics of the two groups of patients and samples.

	Age (years)	Size (mm)	Volume (mL)
Simple cyst (*N* = 13)			
Mean (SD)	39,8 (13,5)	69,54 (14,02)	69,85 (59,36)
Range	20–64	49–98	20–248

Endometrioma (*N* = 14)			
Mean (SD)	38,6 (14,1)	73,71 (33,26)	112,50 (111,77)
Range	28–84	42–170	20–410

The size represents the largest cystic diameter measured on ultrasound. The volume represents the total aspirated volume of intracystic fluid (SD: standard deviation).
